# CaMKII Is Essential for the Function of the Enteric Nervous System

**DOI:** 10.1371/journal.pone.0044426

**Published:** 2012-08-31

**Authors:** Na Gao, Jialie Luo, Karen Uray, Aihua Qian, Shijin Yin, Guodu Wang, Xiyu Wang, Yun Xia, Jackie D. Wood, Hongzhen Hu

**Affiliations:** 1 Department of Integrative Biology and Pharmacology, University of Texas Health Science Center at Houston, Houston, Texas, United States of America; 2 Department of Pediatric Surgery, University of Texas Health Science Center at Houston, Houston, Texas, United States of America; 3 Department of Physiology and Cell Biology, College of Medicine, The Ohio State University, Columbus, Ohio, United States of America; 4 Department of Anesthesiology, College of Medicine, The Ohio State University, Columbus, Ohio, United States of America; University of Houston, United States of America

## Abstract

**Background:**

Ca^2+^/calmodulin-dependent protein kinases (CaMKs) are major downstream mediators of neuronal calcium signaling that regulate multiple neuronal functions. CaMKII, one of the key CaMKs, plays a significant role in mediating cellular responses to external signaling molecules. Although calcium signaling plays an essential role in the enteric nervous system (ENS), the role of CaMKII in neurogenic intestinal function has not been determined. In this study, we investigated the function and expression pattern of CaMKII in the ENS across several mammalian species.

**Methodology/Principal Findings:**

CaMKII expression was characterized by immunofluorescence analyses and Western Blot. CaMKII function was examined by intracellular recordings and by assays of colonic contractile activity. Immunoreactivity for CaMKII was detected in the ENS of guinea pig, mouse, rat and human preparations. In guinea pig ENS, CaMKII immunoreactivity was enriched in both nitric oxide synthase (NOS)- and calretinin-containing myenteric plexus neurons and non-cholinergic secretomotor/vasodilator neurons in the submucosal plexus. CaMKII immunoreactivity was also expressed in both cholinergic and non-cholinergic neurons in the ENS of mouse, rat and human. The selective CaMKII inhibitor, KN-62, suppressed stimulus-evoked purinergic slow EPSPs and ATP-induced slow EPSP-like response in guinea pig submucosal plexus, suggesting that CaMKII activity is required for some metabotropic synaptic transmissions in the ENS. More importantly, KN-62 significantly suppressed tetrodotoxin-induced contractile response in mouse colon, which suggests that CaMKII activity is a major determinant of the tonic neurogenic inhibition of this tissue.

**Conclusion:**

ENS neurons across multiple mammalian species express CaMKII. CaMKII signaling constitutes an important molecular mechanism for controlling intestinal motility and secretion by regulating the excitability of musculomotor and secretomotor neurons. These findings revealed a fundamental role of CaMKII in the ENS and provide clues for the treatment of intestinal dysfunctions.

## Introduction

The Ca^2+^/calmodulin (CaM)-dependent protein kinase II, also known as CaM kinase II or CaMKII, is an important downstream effector of calcium- and calmodulin-mediated signaling pathways [1]. The enzyme has 8–12 isoforms ranging from 52 kDa (α) to 58–61 kDa (β, γ and δ). Both γ and δ isoforms are expressed in all tissues whereas the α and β isoforms are abundantly expressed in the nervous system [1]. In fact, CaMKII makes up nearly 2% of total protein in certain brain regions where it is enriched in postsynaptic densities (PSD), the cytoskeletal specializations on the postsynaptic membrane of excitatory synapses [2], [3].

In the presence of calcium and calmodulin, the enzyme is autophosphorylated on threonine 286 (T^286^) and becomes biologically active [2], [4]. Autophosphorylation of CaMKII leads to translocation of the enzyme to the PSD fractions [2], [4] and upon dephosphorylation it dissociates back to the soluble fraction [2], [4]. Autophosphorylation and activation of the α isoform of CaMKII lead to phosphorylation of glutamate receptors which are essential to learning and memory [2], [3]. CaMKIIα knockout mice display behavioral abnormalities that include decreased fear response and increased defensive aggression, as well as a decrease in serotonin release in putative serotonergic neurons of the dorsal raphe [5].

In addition to neural proteins, CaMKII phosphorylates Ca^2+^-ATPase and phospholamban and affects the function of cardiac, skeletal, and smooth muscle cells [6], [7]. In the gastrointestinal (GI) tract, CaMKII plays important roles in regulating the excitability of intestinal smooth muscle cells (SMCs), thereby influencing gastrointestinal motility [6]. The myogenic effect of CaMKII is mediated by CaMKIIγ and δ which are abundantly expressed by intestinal SMCs [6]. Enhanced activation of CaMKII in intestinal SMCs also contributes to the dysmotility of intestinal SMCs during chemical-induced colitis [8].

It has also been postulated that protein phosphorylation plays a key role in regulating the function of the enteric nervous system (ENS), the “little brain” in the gut [9]. The presence of CaMKII, protein kinase C (PKC), and cyclic AMP-stimulated protein kinase in isolated myenteric ganglia was revealed by using biochemical and immunochemical techniques [10], [11]. A recent report suggests that luminal glucose can induce CaMKII phosphorylation in enterochromaffin cells as well as intrinsic and extrinsic neurons to regulate GI function [12].

Our previous studies have identified CaMKII as an important mediator of neurogenic responses induced by inflammatory mediators such as bradykinin and prostaglandins in the ENS [13]. However, the function and expression profile of CaMKII in the ENS are not fully understood. Important questions are whether CaMKII is enriched in a subset of enteric neurons, whether CaMKII is involved in metabotropic neurotransmissions in the ENS, and whether CaMKII is required for neurogenic regulation of intestinal motility. To answer these questions we investigated the unique distribution pattern and function of CaMKII in the ENS among multiple mammalian species.

## Results

### CaMKII is Expressed by the Inhibitory Motor Neurons in the Myenteric Plexuses of Several Mammalian Species

**Figure 1:**
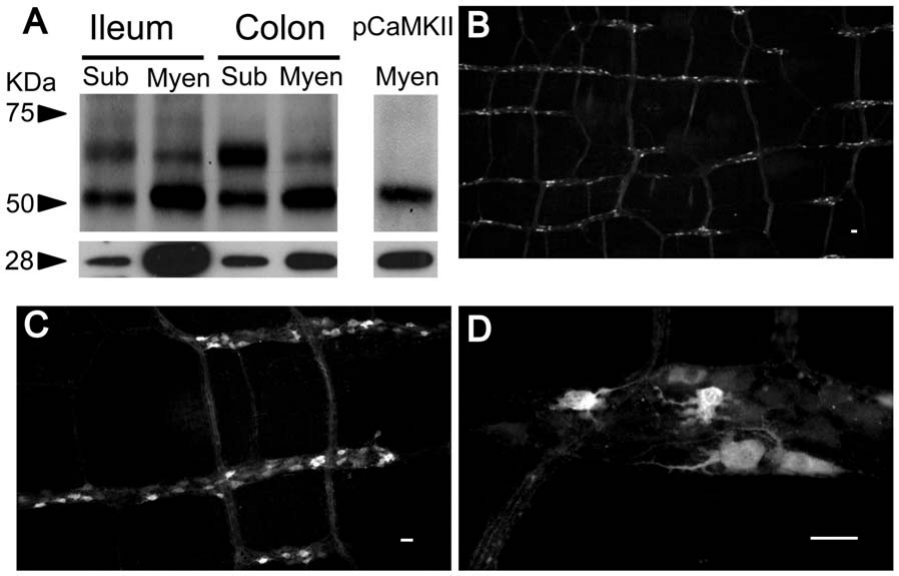
CaMKII is present in the guinea pig ENS. (A) Representative Western Blot analysis reveals the presence of CaMKII-IR in protein extracts of guinea pig myenteric and submucosal plexuses of both ileum and colon. p-CaMKII-IR was also present in the myenteric plexus. Calbindin (28 kD) which is only expressed by enteric neurons was used as a positive control. (B & C) Low magnification images illustrate that many guinea pig ileal myenteric neurons had CaMKII-IR. (D) CaMKII-IR was exclusively expressed by uniaxonal S-type myenteric neurons and present in both cell soma and nerve fibers. Bar = 20 μm.

**Figure 2:**
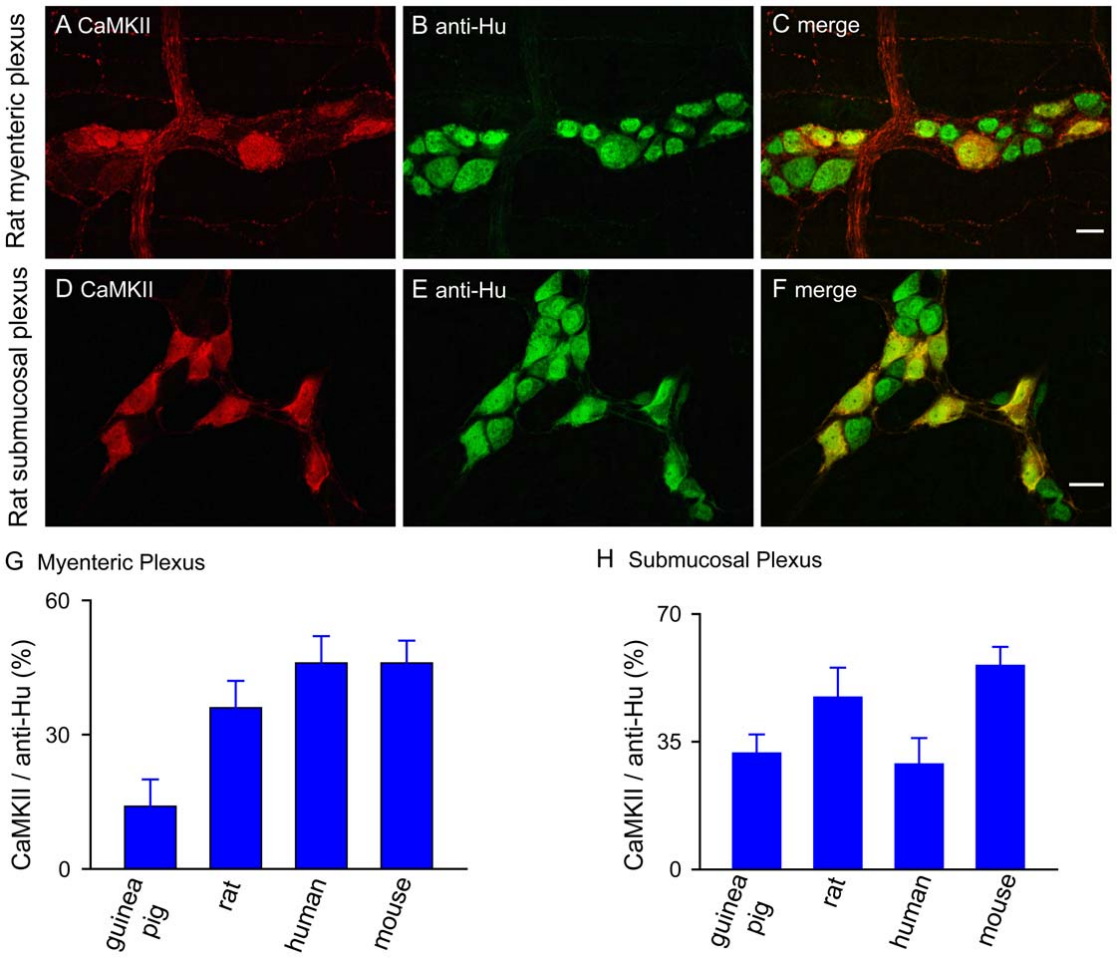
CaMKII is expressed by a subset of neurons in the ENS across multiple mammalian species. (A-C) Representative images illustrate that CaMKII-IR was localized in a subpopulation of rat myenteric neurons. Anti-Hu immunoreactivity was used to label all neurons. (D-F) Representative images show that CaMKII-IR was present in a subpopulation of anti-Hu-immunoreactive rat submucosal neurons. (G) The proportions of CaMKII-immunoreactive neurons in the myenteric plexus of multiple mammalian species. (H) The proportions of CaMKII-immunoreactive neurons in the submucosal plexus across multiple mammalian species.

To reveal the presence of CaMKII in the ENS, we used Western Blot and immunofluorescent staining on enteric neural tissues from multiple species. The presence of CaMKII in the ENS of guinea pig was revealed in total protein isolated from both myenteric and submucosal plexuses of ileum and colon by Western Blot (Figure 1A). We used calbindin as a positive control. Two major bands were present, one corresponded to CaMKIIα (≈52 kDa) and the other to CaMKIIβ, γ and δ isoforms (58–61 kDa). The intensity of the 52 kDa band was much higher in the myenteric plexus of both ileum and colon, presumably reflecting that CaMKIIα is the predominant isoform in guinea pig myenteric plexus. We also detected the presence of autophosphorylated CaMKII (p-CaMKII) in the myenteric plexus without stimulation, suggesting that the p-CaMKII is present in the myenteric plexus at the basal state (Figure 1A). In guinea pig myenteric plexus CaMKII immunofluorescent staining showed that CaMKII-immunoreactivity (IR) was evident in neurons and nerve fibers (Figure 1B-1D). All CaMKII-immunoreactive neurons had uniaxonal (Dogiel type I) morphology (Figure 1D). As expected, CaMKII-immunoreactivity was abundantly distributed in the ENS across all species tested including guinea pigs, rats, humans and mice. The proportions of CaMKII-positive neurons among different species are between 14 to 46% in the myenteric plexus and 29 to 56% in the submucosal plexus as determined by double-labeling of CaMKII-IR with anti-Hu immunoreactivity which is displayed by all enteric neurons [14]–[15] (Figure 2).

**Figure 3:**
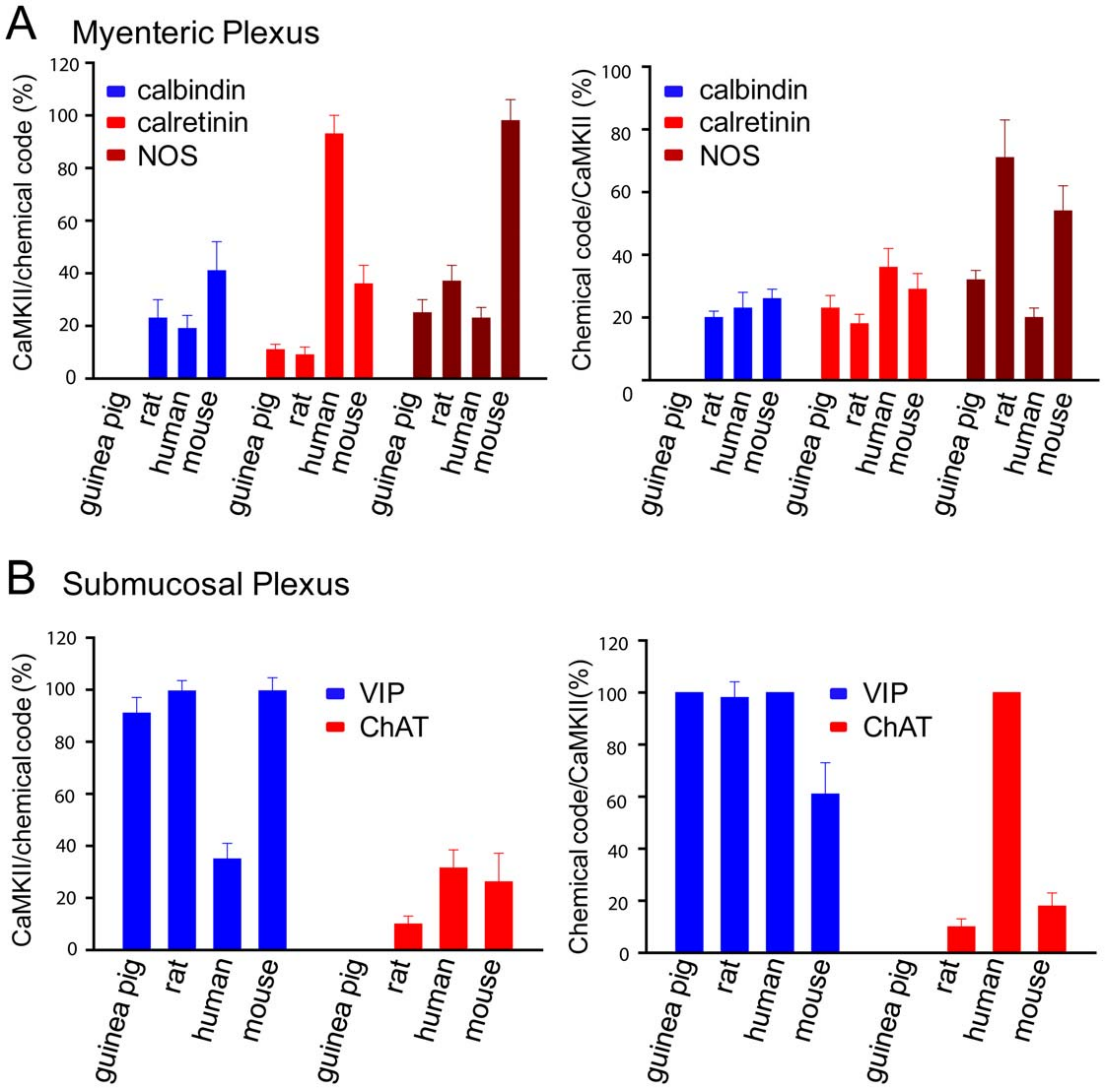
Distribution of CaMKII in relation to chemical codes in both myenteric (A) and submucosal (B) plexuses of ileum among different mammalian species.

**Figure 4:**
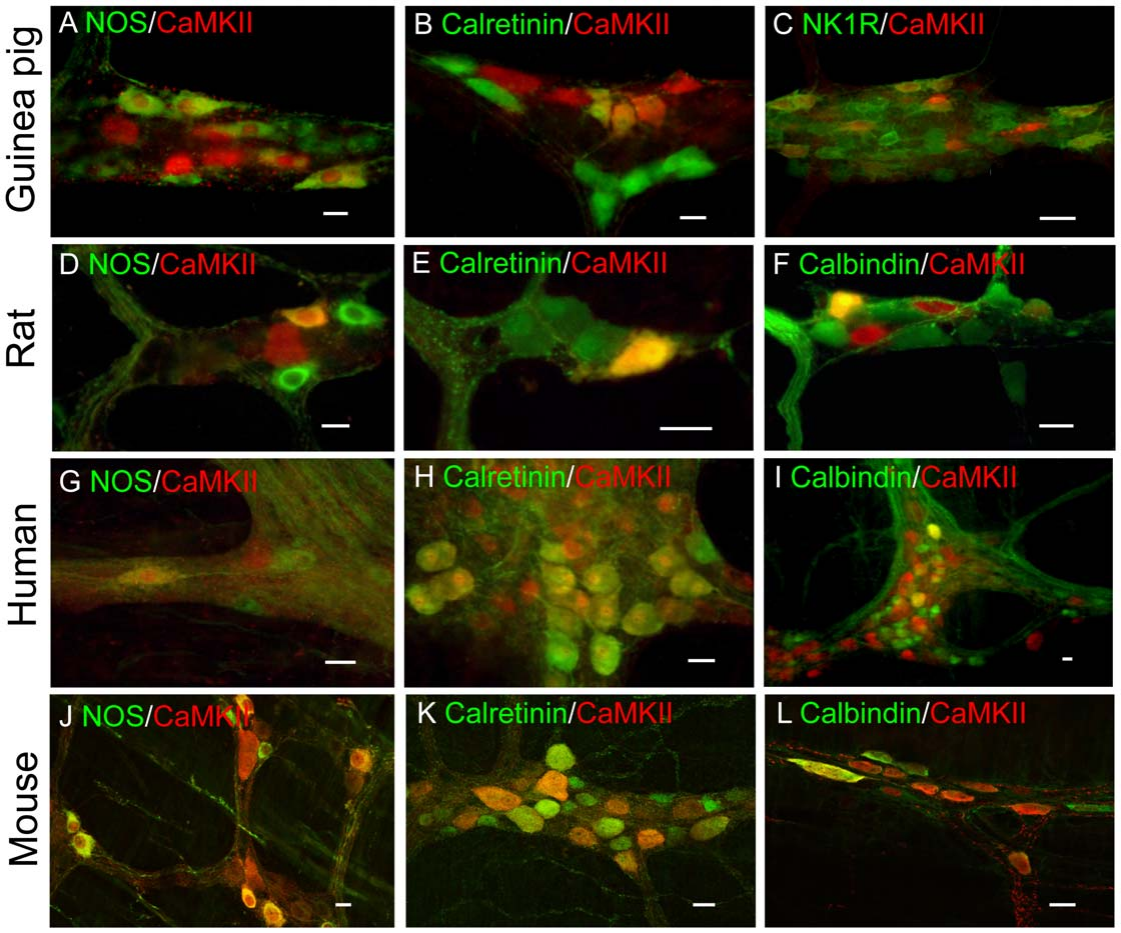
Localization and chemical coding of CaMKII in the ileal myenteric plexuses of guinea pig, rat, human and mouse. In guinea pig ileal myenteric plexus CaMKII-IR colocalized with NOS- (A), calretinin- (B) and NK1R- (C) IR. CaMKII-IR also colocalized with NOS- (D), calretinin - (E) and calbindin - (F) IR in rat ileal myenteric plexus. In human duodenal myenteric plexus CaMKII-IR colocalized with NOS- (G), calretinin- (H) and calbindin- (I) IR. In mouse ileal myenteric plexus CaMKII-IR also colocalized extensively with NOS- (J), calretinin- (K) and calbindin- (L) IR. Yellow color refers to overlapping images. Bar = 20 μm for all images.

We next used double-labeling technique to identify neurochemically the CaMKII-immunoreactive neurons in the ENS of multiple mammalian species including guinea pigs, rats, humans and mice in order to gain a better understanding of neurons associated with CaMKII-IR. Distribution of CaMKII in relation to chemical codes in the ENS of multiple mammalian species is shown in Figure 3. In the guinea pig ileum, the best-characterized region of the ENS [16], [17], nearly 25% of the NOS-immunoreactive neurons expressed CaMKII-IR in the myenteric plexus. About 32% of the total CaMKII-immunoreactive neurons contained NOS-IR (Figure 4A). About 23% of CaMKII-immunoreactive neurons also expressed calretinin-IR and about 11% of total calretinin-immunoreactive neurons contained CaMKII-IR (Figure 4B). About 14% of CaMKII-immunoreactive neurons expressed NK1R-IR and about 17% of the total NK1R-immunoreactive neurons contained CaMKII-IR (Figure 4C). By contrast, _ENREF_28immunoreactivity for CaMKII and calbindin was identified in distinct groups of myenteric neurons and no overlapping was detected (Figure S1). No CaMKII-IR was colocalized with NPY-IR, a neuronal marker for descending interneurons or motor neurons most of which are associated with NOS-IR [16] [18](Figure S1).

Thirty-seven percent of the CaMKII-immunoreactive neurons in rat myenteric plexus had NOS-IR and about 70% of NOS-immunoreactive neurons had CaMKII-IR (Figure 4D). In contrast to guinea pig myenteric plexus, about 20% of the calbindin-immunoreactive neurons in the rat myenteric plexus had CaMKII-IR and about 23% of CaMKII-immunoreactive neurons expressed calbindin-IR (Figure 4E). Nine percent of CaMKII-immunoreactive neurons also contained calretinin-IR and about 18% of calretinin-immunoreactive neurons expressed CaMKII-IR (Figure 4F). Please note that CaMKII-immunoreactive neurons associated with calbindin- or calretinin-IR had uniaxonal (Dogiel type I) morphology in the rat myenteric plexus (Figure 4E & 4F).

CaMKII-IR was also abundant in human duodenal myenteric neurons. Twenty percent of the CaMKII-immunoreactive neurons expressed NOS-IR and 23% of the NOS-immunoreactive neurons expressed CaMKII-IR (Figure 4G). Thirty-six percent of CaMKII-immunoreactive neurons expressed calretinin-IR and 93% of the calretinin-immunoreactive neurons expressed CaMKII-IR (Figure 4H). About 23% of the CaMKII-immunoreactive neurons were immunoreactive for calbindin and 19% of the calbindin-immunoreactive neurons expressed CaMKII-IR (Figure 4I).

In mouse ileum, 98% of NOS-immunoreactive myenteric neurons were immunoreactive with CaMKII, while 54% of CaMKII-immunoreactive neurons were double labeled with NOS-IR (Figure 4J). Of calretinin-immunoreactive neurons, 36% were immunoreactive for CaMKII, and 29% of CaMKII-immunoreactive neurons were immunoreactive for calretinin (Figure 4K). Approximately 41% of the calbindin-immunoreactive myenteric neurons in mouse ileum were immunoreactive for CaMKII and about 26% of CaMKII-immunoreactive neurons were double labeled with calbindin-IR (Figure 4L). These results suggest that CaMKII is enriched in both cholinergic and non-cholinergic myenteric neurons across different mammalian species.

### CaMKII is Expressed by the Submucosal Secretomotor Neurons of Several Mammalian Species

**Figure 5:**
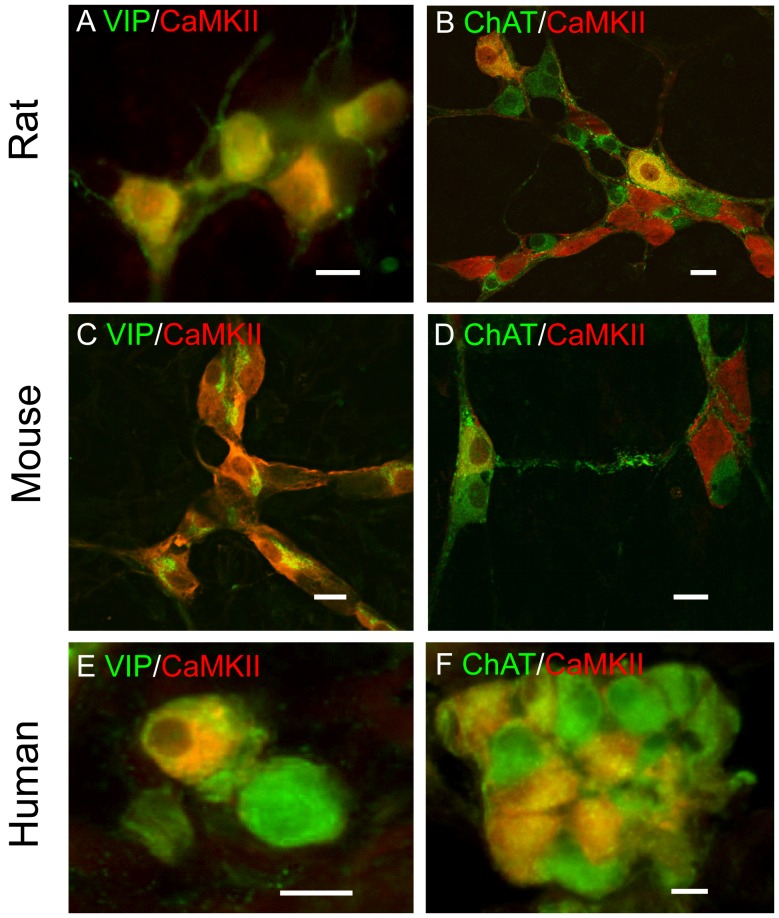
Localization and chemical coding of CaMKII in rat, human and mouse submucosal plexuses. CaMKII-IR was localized in almost all VIP-immunoreactive neurons (A) and some ChAT-immunoreactive neurons (B) in rat submucosal plexus. In the mouse submucosal plexus, CaMKII-IR also colocalized extensively with VIP-IR (C) but much less with ChAT-IR (D). On the other hand, CaMKII-IR colocalized with both VIP-IR (E) and ChAT-IR (F) in human submucosal plexus. Yellow color refers to overlapping images. Bar = 20 μm for all images.

Our previous studies found that CaMKII in guinea pig submucosal plexus was exclusively localized in noncholinergic/vasodilator secretomotor neurons (i.e. VIPergic neurons) and is required for the excitatory action of the inflammatory mediator bradykinin [13]. We investigated whether the expression pattern of CaMKII in the submucosal plexus is conserved among mammals by double-labeling CaMKII and other “chemical codes” in the submucosal plexus of rat, mouse and human. In rat submucosal plexus, 98% of the CaMKII-immunoreactive neurons expressed VIP and nearly all VIP-immunoreactive neurons expressed CaMKII (Figure 5A). On the other hand, only about 10% of ChAT-immunoreactive neurons expressed CaMKII-IR and about 10% of CaMKII-immunoreactive neurons expressed ChAT-IR (Figure 5B). Consistent with being present in the rat cholinergic submucosal neurons, CaMKII-IR was also colocalized with both cholinergic neuronal markers calbindin and calretinin (Figure S2).

In mouse submucosal plexus, almost all VIP-immunoreactive neurons (99%) were labeled with CaMKII-IR and only about 26% of the ChAT-immunoreactive neurons were immunoreactive for CaMKII. About 61% of CaMKII-immunoreactive neurons expressed VIP-IR and 18% contained ChAT-IR (Figure 5C & 5D). CaMKII-IR was also present in both calbindin- and calretinin-immunoreactive neurons in mouse submucosal plexus (Figure S2).

In human submucosal plexus, all CaMKII-immunoreactive neurons expressed VIP and 35% of the VIP-immunoreactive neurons contained CaMKII-IR (Figure 5E). Unlike rats, guinea pigs and mice, many VIP-immunoreactive submucosal neurons also express ChAT in humans [19]. Accordingly, all CaMKII-immunoreactive neurons also expressed ChAT-IR and 32% of the ChAT-immunoreactive neurons contained CaMKII-IR (Figure 5F).

Taken together, these data suggest that CaMKII is expressed by both cholinergic and peptidergic submucosal neurons but CaMKII-IR is associated with variable proportions of cholinergic neurons among different species.

### CaM and CaMKII Mediate the Slow Excitatory Postsynaptic Potentials (EPSPs) in the ENS

**Figure 6:**
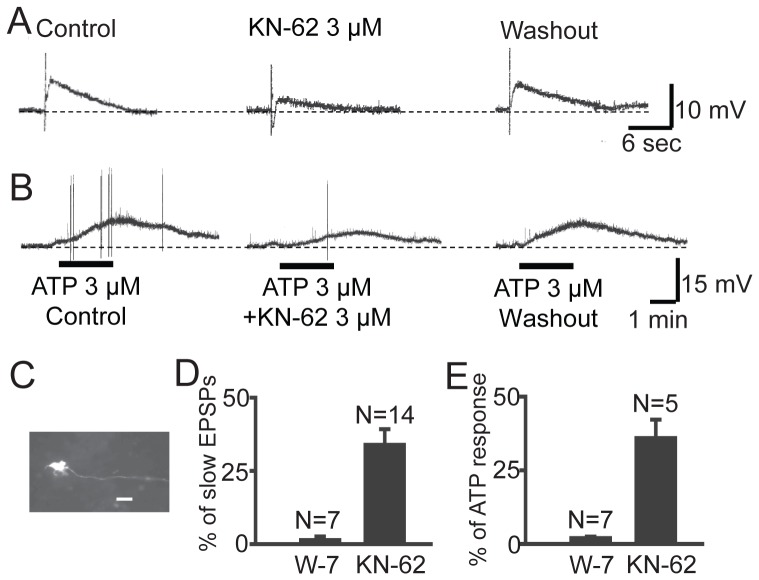
CaMKII is required for metabotropic purinergic signaling in the ENS. (A) Slow EPSPs were evoked by electrical stimulation to the interganglional fiber tract in guinea pig submucosal plexus. The CaMKII inhibitor KN-62 suppressed the slow EPSP in a reversible manner. (B) KN-62 also suppressed ATP-induced slow EPSP-like response in the same cell as shown in A. (C) The uniaxonal morphology of the recorded cells was revealed by staining biocytin injected into the cell through microelectrode. (D) Summary statistics of the effect of W-7 and KN-62 on stimulus-evoked slow EPSPs. (E) Summary statistics of the effect of W-7 and KN-62 on ATP-induced slow EPSP-like response.

Although calcium signaling plays a critical role for the normal function of the ENS [20], [21], the role of CaM and CaMKII in enteric neuronal signaling is not fully understood. Our previous studies have found that inhibitors of CaM and CaMKII suppressed excitatory neuronal response evoked by inflammatory mediators such as bradykinin and prostaglandins in guinea-pig submucosal plexus [13], suggesting that CaM/CaMKII signaling is essential to the function of metabotropic G-protein coupled receptors (GPCRs) in the ENS. Multiple GPCRs are involved in slow EPSPs in the ENS including the P2Y1 receptor and the NK1 receptor [22], [23], [24]. Since both NK1 and the P2Y1 receptors belong to the Gq-coupled protein receptor family which coupled to phospholipase C and intracellular calcium mobilization, we hypothesized that CaM-CaMKII signaling is involved in the slow synaptic transmission in ENS. We tested this hypothesis with intracellular recordings in the guinea pig submucosal plexus where the purinergic slow EPSPs are present in nearly 83% of secretomotor neurons [23], [25]. As expected, MRS2179, a selective antagonist for the P2Y1 receptor, suppressed or abolished stimulus-evoked slow EPSPs in 30 out of 34 submucosal neurons examined (not shown). The membrane-permeable CaM inhibitor W-7 was used to test the hypothesis that Ca^2+^ sensing by CaM is involved in the P2Y1 receptor-mediated signal transduction cascade. Application of W-7 did not alter the resting membrane potential of the neurons. Pretreatment with W-7 (50 µM) for 15 min reduced the amplitude of the stimulus-evoked purinergic slow EPSP to 1.4±1.2% (P<0.01, paired t test) of the value prior to W-7 application in 7 neurons (Figure 6D). In 5 of the 7 neurons, W-7 abolished the purinergic slow EPSPs. The effect of W-7 on responses to 3 µM ATP was also determined in view of mediation of the slow EPSP by ATP. W-7 (50 µM) in the bathing solution suppressed the amplitude of ATP-evoked depolarizing responses to 2.0±0.5% (P<0.01, paired *t* test) of the value prior to W-7 application in 7 neurons (Figure 6E). Washout of W-7 for a minimum of 45 min reversed its inhibitory action on responses to ATP as well as on the slow EPSPs (not shown).

A specific CaMKII inhibitor, KN-62 was used as a pharmacological tool for study of the involvement of CaMKII in the intraneuronal signal transduction pathway for the purinergic slow EPSPs and ATP-induced slow EPSP-like responses (Figure 6A & 6B). Application of 3 µM KN-62 did not alter the resting membrane potential of the neurons. After 30 min treatment with 3 µM KN-62, the mean amplitude of the slow EPSPs evoked by a single pulse of electric shock to the interganglional fiber tract was reduced to 33.9±5.3% (P<0.01, paired t test) of the value prior to KN-62 treatment in 14 neurons (Figure 6A & 6D). Washout of KN-62 for a minimum of 45 min reversed its inhibitory action on the slow EPSPs. Addition of KN-62 (3 µM) in the bathing solution also suppressed the amplitude of ATP-evoked depolarizing responses to 34.9±6.3% (P<0.01, paired *t* test) of the value prior to the addition of KN-62 to the tissue bath in 5 neurons (Figure 6B & 6E). These results suggest that CaM-CaMKII signaling is required for the purinergic slow synaptic transmissions in the ENS. Since single electric pulses generally evoke the purinergic slow EPSPs while trains of pulses can evoke slow EPSPs mediated by receptors other than P2Y1 receptors, we also tested the effect of KN-62 on slow EPSPs evoked by a train of electrical stimuli (4 shocks, 150–200 Hz). Similar to that induced by single pulses, KN-62 (3 µM) suppressed the slow EPSPs evoked by trains of stimulations in a reversible manner (n = 3, Figure S3). Therefore, it is likely that CaMKII signaling is also involved in non-purinergic slow EPSPs.

### CaMKII is Required for Neurogenic Inhibition of Intestinal Motility

A fundamental function of the ENS is to generate the major patterns of contractile motor behavior found in the intestine and colon [26]. Previous studies have found that the ENS exerts a tonic inhibitory brake on myogenic activity of the intestinal circular muscle coat, as revealed by increased amplitude of contractile responses to each and every electrical slow-wave after blockade the activity of inhibitory musculomotor neurons by tetrodotoxin (TTX) [27], [28]. TTX does not generally affect myogenic contraction in the GI tract as TTX fails to abolish action potential generation in intestinal SMC [29]. Therefore, TTX is a useful pharmacological tool for distinguishing direct action on SMC from indirect actions mediated by intrinsic nerves and can be used as readout of the neurogenic inhibition of intestinal SMC by the ENS. Nevertheless, the intraneuronal mechanisms that generate the constitutive firing of inhibitory musculomotor neurons are unknown.

**Figure 7:**
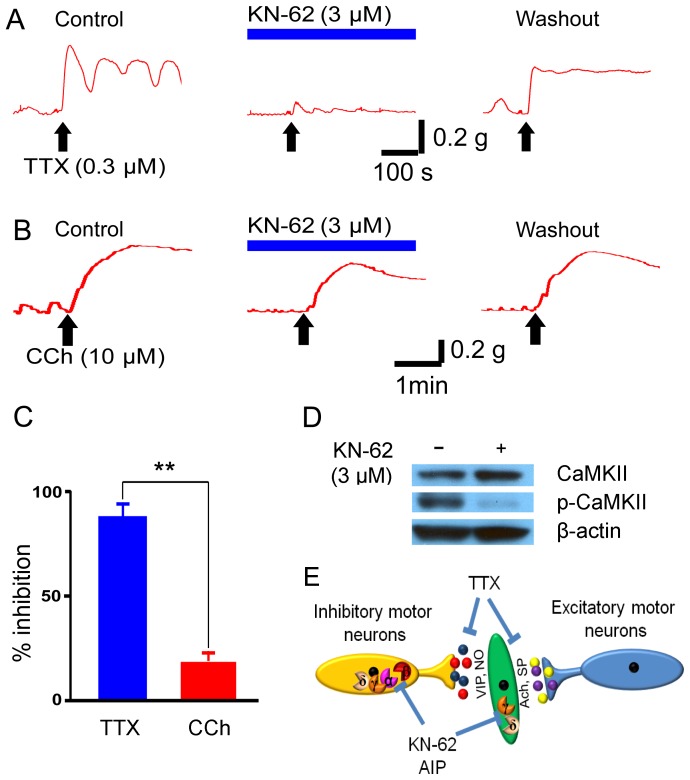
CaMKII is required for neurogenic inhibition of colon motility. (A) KN-62 nearly abolished TTX-evoked contractile response in a mouse colon strip. The effect of KN-62 was partially reversible after about 1 hr washout. (B) Bath application of KN-62 also suppressed CCh-induced myogenic contraction in a reversible manner. (C) The inhibitory effect of KN-62 on TTX-induced response was significantly stronger than on CCh-induced contraction. ** p<0.01. (D) Representative Western Blot analysis shows that pretreatment with 3 µM KN-62 for 15 min dramatically decreased the p-CaMKII-IR in the myenteric plexus preparations of mouse colon. The total CaMKII-IR was comparable between tissue preparations with and without treatment with KN-62. β-actin was used as a loading control. (E) Schematic diagram illustrates the neurogenic and myogenic roles of CaMKII in controlling the colonic intestinal motility.

Considering that CaMKII is abundantly distributed in myenteric inhibitory musculomotor neurons across multiple species and that CaMKII is involved in the signal transduction pathways required for normal synaptic transmission in the ENS, we hypothesized that CaMKII in inhibitory musculomotor neurons is involved in generation of spontaneous firing and ongoing release of inhibitory transmitters at neuromuscular junctions in the intestinal circular muscle coat. We tested this hypothesis by investigating the effects of KN-62 on the action of TTX to suppress excitability of inhibitory musculomotor neurons and remove ongoing braking action of spontaneously inhibitory musculomotor neurons on contractile activity of mouse colonic muscle strips. We chose to use mouse colon strips because previous studies show that TTX causes contraction of the mouse colon but mouse stomach and small intestine are less sensitive to TTX [27]. Furthermore, we found that nearly all NOS-immunoreactive myenteric neurons had CaMKII-IR (not shown). As expected, blocking neurotransmissions by TTX consistently evoked contractile responses in all 19 colonic strips examined as revealed by either increased frequency of spontaneous activity or elevation of baseline tension (Figure 7A). Pretreatment with KN-62 (3 µM, 5–15 min) suppressed or abolished TTX-induced contractile response, suggesting that CaMKII activity is required for spontaneous firing of inhibitory musculomotor neurons (Figure 7A & 7C). The effect of KN-62 was long-lasting, requiring up to 50 min for partial recovery of ability of TTX to increase baseline tension and elevated amplitude of spontaneously-occurring contractions (Figure 7A). Western Blot analysis confirmed that treatment with KN-62 (3 µM, 15 min) dramatically reduced p-CaMKII-IR in the myenteric plexus of mouse colon but the total CaMKII-IR was comparable to that without KN-62 treatment (Figure 7D).

Previous studies have found that CaMKII γ and δ isoforms are abundantly expressed by intestinal SMCs [30]. Suppression of CaMKII activity by KN-62, at 1.5 to 3 µM, reduced carbachol (CCh)-induced contractile response in both proximal and distal colon by 20.6±3.9% of the (P<0.01, paired t test, n = 5). The results support the hypothesis that CaMKII is involved in the postreceptor signal transduction of the muscarinic receptor-mediated colonic muscle contraction [6], [30]. Nevertheless, suppression by KN-62 of TTX-induced increase in contractile activity was significantly stronger than that of CCh-evoked muscle contraction (P<0.01, student t test, n = 9). Interestingly, KN-62 (3 µM) alone also increased the colonic spontaneous activity in a proportion of colon strips (4 out of 24), suggesting that suppression of CaMKII alone is sufficient to attenuate the neurogenic inhibitory outflow to the intestinal SMCs (Figure S4). In 4 out of 14 colon strips we found that KN-62 reversed TTX-induced contractile response to relaxation of tension (Figure S4).

We also tested autocamtide-2-related inhibitory peptide (AIP), which is another well-established cell-permeable selective CaMKII inhibitor. Pre-incubation with AIP (1 µM, 5–15 min) suppressed TTX-induced contractile response in mouse colon and the effect of AIP on TTX-induced contractile response was significantly stronger than that on CCh-induced contraction (P<0.01, student t test, n = 5) (Figure S5). AIP effect increased with time with a slow onset when applied 5 to 15 min before addition of TTX or CCh. The suppression of p-CaMKII by treatment with AIP (1 µM, 15 min) was confirmed by Western Blot analysis (Figure S5).

These results strongly suggest that CaMKII in the myenteric inhibitory motor neurons is part of the mechanism that mediates the tonic neurogenic inhibition of intestinal SMC and CaMKII is required to drive the enteric inhibitory motor neurons into a constitutively active state.

## Discussion

Our study provides evidence that CaMKII involvement in intracellular signal transduction in ENS is conserved across multiple mammalian species. It is widely expressed by neurons in both myenteric and submucosal plexuses including the myenteric inhibitory musculomotor neurons that innervate the intestinal circular muscle coat and submucosal VIPergic secretomotor neurons that control intestinal secretion in multiple mammalian species [16], [17]. CaMKII activity is required for metabotropic synaptic transmissions and suppression of CaMKII activity leads to a decrease in synaptic strength in the ENS. CaMKII is also required for cross-talk between the myenteric neurons and intestinal SMC to generate and/or maintain the inhibition of autogenic excitability of the musculature in continuous manner. The results support the hypothesis that CaMKII is not only a key player in the neural plasticity in the central nervous system (CNS) but also critically involved in physiology and pathophysiology of the “little brain” in the gut.

### CaMKII in Inhibitory Musculomotor Neurons

CaMKII is expressed by diverse subpopulations of myenteric plexus neurons of all species. Orally projecting calretinin-immunoreactive interneurons express CaMKII among different species, especially in the human myenteric plexus. On the other hand, “chemical coding” of CaMKII in the myenteric plexus is also variable among species examined, for instance, CaMKII-IR is not found in the calbindin-expressing myenteric neurons in guinea pig [13] but about 20% of the calbindin-immunoreacetive myenteric neurons in other mammalian species tested have CaMKII (Figure 3). CaMKII is also colocalized substantially with NOS, the chemical codes for the inhibitory motor neurons with descending projections to the musculature [16], [17]. The presence of CaMKII in both non-cholinergic and cholinergic enteric neurons suggests that CaMKII has diverse functions in the ENS.

Pools of inhibitory musculomotor neurons to the circular muscle discharge continuously and produce constant inhibition of the autogenic activity of the circular muscle. NO and VIP are major inhibitory neurotransmitters released at neuromuscular junctions along the gastrointestinal tract. When the inhibitory neuronal discharge is blocked by TTX, every electrical slow-wave cycle triggers intense discharge of action potentials and large-amplitude contractions [27], [28]. The presence of CaMKII in NOS-containing myenteric inhibitory motor neurons indicates that activation of CaMKII is likely to have consequences on intestinal motility which is tonically regulated by ENS. Indeed, inhibition of CaMKII by KN-62 and AIP abolished the TTX-induced contractile response in mouse colon. Although inhibition of CaMKII also suppressed about 20% of the CCh-induced contractile response, it nearly abolished TTX-evoked contractile response. Therefore, neurogenic CaMKII signaling plays a dominant role in regulating mouse colonic motility. Our results suggest that CaMKII activation is a step in one of the intraneuronal signal transduction cascades operating in ENS neurons that have Dogiel Type I uniaxonal morphology and S-type electrophysiological and synaptic behavior [13], [31]. The spontaneous-ongoing discharge of inhibitory musculomotor neurons is dependent upon actively working CaMKII.

Interestingly, CaMKII is abundantly expressed by neurons associated with NK1R-IR in guinea pig myenteric plexus. As NK1R is a mediator of slow EPSPs in the NOS-positive inhibitory motor neurons, it is tempting to speculate that CaMKII might also be involved in NK1R-mediated slow synaptic transmission in guinea pig myenteric plexus [32], [33], [34]. Whether NK1R is involved in CaMKII-mediated spontaneous-ongoing discharge of inhibitory musculomotor neurons requires further investigation.

In a proportion of colon strips the CaMKII inhibitor KN-62 increases the spontaneous contractile response which is reminiscent of TTX-evoked increase of the colonic spontaneous response. Since the intestinal spontaneous activity is regulated by both excitatory and inhibitory motor neurons, the selective loss of CaMKII activity in the inhibitory but not the excitatory motor neurons might contribute to the excitatory action evoked by KN-62 as well as TTX-induced decrease of muscle tension in the presence of KN-62 (Figure 7E, Figure S4).

The intrinsic CaMKII in the intestinal SMCs plays an important role in regulating intestinal contractility [6], [8], [11], [35]. It was reported that a selective CaMKII inhibitor KN-93 has differential effects on SMCs in the fundus and colon, i.e. enhancement of ACh-induced fundus contractions but inhibition of proximal colon contractions. We also confirmed that both KN-62 and AIP suppressed carbachol-induced colonic contraction. Therefore, it seems that the CaMKII-mediated neurogenic and myogenic activities have opposite effects on the colonic motility, i.e. direct increase of excitability of SMCs and indirect inhibition of the SMC excitability by promoting release of the inhibitory neurotransmitters from the myenteric inhibitory motor neurons. This is also supported by our observation that inhibition of CaMKII by KN-62 or AIP did not induce robust contraction in all colon strips because both CaMKII-mediated myogenic contraction and neurogenic relaxation were blocked concomitantly. Under this condition, TTX could no longer induce neurogenic contractions because CaMKII inhibitors had already blocked the function of inhibitory musculomotor neurons.

Although ICC is an important part of the electromechanical coupling machinery in the gut, it is unlikely that ICC plays a direct role in KN-62-mediated response because CaMKII is not present in ICC [36]. However, ICC could still be a vital part of the mechanisms that convey the neurogenic action of CaMKII to the intestinal SMCs [37].

### CaMKII in Secretomotor Neurons

In the submucosal plexus, CaMKII is extensively expressed by VIPergic secretomotor neurons among all species. Please note that CaMKII is also expressed by most ChAT-positive submucosal neurons in humans. This is because that many VIP-immunoreactive submucosal neurons also express ChAT in humans [19]. The function of CaMKII in the VIPergic secretomotor neurons is further confirmed by intracellular recording experiments in guinea pig submucosal plexus where KN-62 suppressed the P2Y1 receptor-mediated slow EPSPs and ATP-induced depolarizing responses which are exclusively recorded in the VIPergic secretomotor neurons [23]. Furthermore, KN-62 also suppressed slow EPSPs evoked by trains of stimulations which most likely involve non-purinergic components. The requirement of CaMKII for the slow excitatory neurotransmissions in the submucosal plexus supports the hypothesis that CaMKII is an important determinant of neurogenic intestinal secretion [38]. This conclusion is further supported by our previous findings that CaMKII plays an important role in purinergic neurogenic intestinal secretion [39]. Nevertheless, cholinergic submucosal neurons in rat, mouse and human also express CaMKII. Therefore, CaMKII might be differentially involved in calcium signaling between cholinergic and non-cholinergic secretomotor neurons among different mammalian species.

Viewed together, these data suggest that CaMKII performs a major function in the ENS by mediating metabotropic synaptic transmission and boosting the neurogenic inhibitory outflow to the intestinal SMCs. The neurogenic action of CaMKII might be part of the mechanism underlying the constitutive neurogenic inhibitory tone in the gastrointestinal tract. The neurogenic actions of CaMKII could also contribute to fundamental mechanisms underlying physiology and pathophysiology of gastrointestinal motility and secretion among mammals. Interestingly, CaMKIIα knockout mice clearly show altered behavioral phenotypes including defects in learning and memory. It is tempting to speculate that the CaMKIIα knockout mice might also display altered gastrointestinal phenotypes, for instance, a decreased CaMKII signaling in the myenteric plexus could result in increased spontaneous intestinal contraction, which could lead to increased GI transit. Further investigations are required to explore these possibilities.

## Materials and Methods

### Tissue Preparations

Tissues were obtained from Hartley-Dawley guinea pigs, C57BL/6 mice and Sprague Dawley rats which were killed by decapitation under isofluorane inhalation. The procedures were approved by the Ohio State University Animal Experimentation Ethics Committee or The Center for Laboratory Animal Medicine and Care (CLAMC) at The University of Texas Health Science Center at Houston. Whole mounts of the myenteric and submucosal plexuses were prepared from these segments as described previously (Methods S1) [13]. Fresh segments of human jejunum discarded during Roux-En-Y gastric bypass surgeries were used after obtaining written informed consent from the patients. Procedures for human studies were approved by the Institutional Biosafety Committee and The Office of Responsible Research Practices of The Ohio State University. Tissues of about 3 cm in length and 2 mm in width were stretched and pinned and fixed in Zamboni’s fixative overnight. The fixed tissues were either dissected for whole mount preparation or immersed in 30% sucrose overnight before preparations of 8 µm frozen sections.

### Western Blot

Membrane proteins were extracted from preparations of guinea pig ileal myenteric and submucosal plexuses, mouse colonic myenteric plexus and 40 µg of protein per lane was resolved by gel electrophoresis followed by transfer to polyvinylidene fluoride transfer membranes (Pall Corporation). Membranes were blocked with 5% nonfat milk in Tris-buffered saline (TBS) with 0.1% Tween 20 (TBST) for 1 hour at room temperature. After being washed with TBST, the membranes were incubated overnight at 4°C with a primary antibody against CaMKII or p-CaMKII (Thr286) (Table S1). After washing, the membranes were incubated for 1 hour at room temperature with horseradish peroxidase-conjugated goat anti-mouse IgG (1∶20,000; Amersham) or goat anti-rabbit IgG (1∶5,000; Amersham). The immunoblots were detected with enhanced chemiluminescence reagents (Amersham).

### Immunofluorescence

Method for immunofluorescent staining of whole-mounts or frozen sections has been described previously (Methods S1) [13]. Primary antibodies used are listed in Table S1. All preparations were examined with an epifluorescence microscope (Nikon Eclipse-1000) or Nikon A1 Confocal Laser Microscope System. Pictures were taken with a CCD digital camera and analyzed in a SPOT III program or NIS-Elements.

### Intracellular Recording

Intracellular “sharp” microelectrode recordings were performed as described previously on guinea pig submucosal plexus (Methods S1) [23]. Briefly, transmembrane electric potentials were recorded using the preamplifier (M767, World Precision Instruments). Synaptic potentials were evoked by focal electrical stimulation of interganglionic fiber tracts. Data were recorded and analyzed using PowerLab data acquisition system (AD Instruments). All chemicals were purchased from Tocris Bioscience. Chemicals were applied by addition to the superfusion solution.

### Intestinal Motility Assay

Intestinal contractile activity was determined on mouse colon strips as previously described [40]. Briefly, 1-cm whole thickness colon strips from the mouse colon of each animal were prepared in the longitudinal direction and mounted in duplicate in 25-ml organ baths filled with Krebs-Ringer solution gassed with 5% CO_2_–95% O_2_. After a 30 min equilibration period, the isometric force was monitored by an external force displacement transducer (Quantametrics) connected to a PowerLab data acquisition system (AD Instruments). Mean contraction amplitude was used for quantification.

### Statistical Methods

Statistically significant differences between means were determined by paired *t*-tests or 2-tailed Student’s *t-*tests. P<0.05 is considered significantly different.

## Supporting Information

Figure S1 **CaMKII-immunoreactive myenteric neurons do not express calbindin or NPY.** (A-C) CaMKII-IR was not colocalized with calbindin-IR. (D-F) CaMKII-IR was not colocalized with NPY-IR in the guinea pig myenteric plexus. Bar = 20 μm.
(TIF)

Figure S2 **CaMKII-IR is co-locolized with both calbindin- (A, C) and calretinin-IR (B, D) in rat and mouse submucosal plexuses.**
(TIF)

Figure S3 **KN-62 suppresses slow EPSPs evoked by train stimulations (4 pulses, 150–200 Hz). KN-62 (3 µM) was applied through the bath solution.** 30 min incubation of KN-62 suppressed the slow EPSP. The effect of KN-62 was reversible manner after a 40 min washout. The recorded neuron shows uniaxonal morphology as revealed by staining of biocytin injected via the recording microelectrode. Bar = 20 µm.
(TIF)

Figure S4 **Application of KN-62 produces contraction or reverses TTX-induced contractile response in mouse colon strips.** (A) Representative trace illustrates that KN-62 causes an increased contractile response in a mouse colon strip. (B) Application of KN-62 reversed TTX-induced contractile response in the same mouse colon strip.
(TIF)

Figure S5 **Suppression of TTX-induced contractile response by the selective CaMKII inhibitor, Autocamtide-2-related inhibitory peptide (AIP) in mouse colon.** (A) AIP (1 µM) suppressed TTX-induced contractile response. The effect of AIP was partially reversible. (B) Effect of AIP on CCh-induced contraction in a colon strip. (C) The inhibitory effect of AIP on TTX-induced contractile response was significantly stronger than on CCh-induced contraction. ** p<0.01. (D) Representative Western Blot analysis shows that pretreatment with 1 µM AIP for 15 min substantially reduced the p-CaMKII-IR in the myenteric plexus preparations of mouse colon. The total CaMKII-IR was comparable between tissue preparations with and without treatment with KN-62. β-actin was used as a loading control.
(TIF)

Table S1 **Antisera and antibodies used.**
(TIF)

Methods S1 (DOCX)
