# Supplementary figures and images for: CaMKII Is Essential for the Function of the Enteric Nervous System
Source: PLoS One. 2012 Aug 31;7(8):e44426. doi: 10.1371/journal.pone.0044426 (PMC3432132; doi:10.1371/journal.pone.0044426)

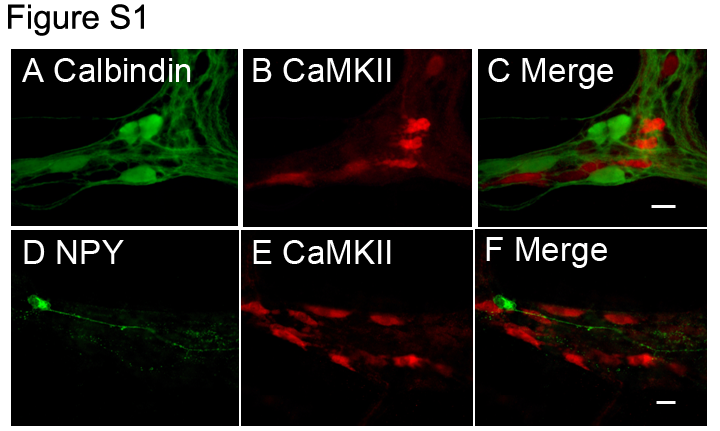

Supplement: Figure S1 — CaMKII-immunoreactive myenteric neurons do not express calbindin or NPY. (A-C) CaMKII-IR was not colocalized with calbindin-IR. (D-F) CaMKII-IR was not colocalized with NPY-IR in the guinea pig myenteric plexus. Bar = 20 μm. (TIF) [file pone.0044426.s001.tif]

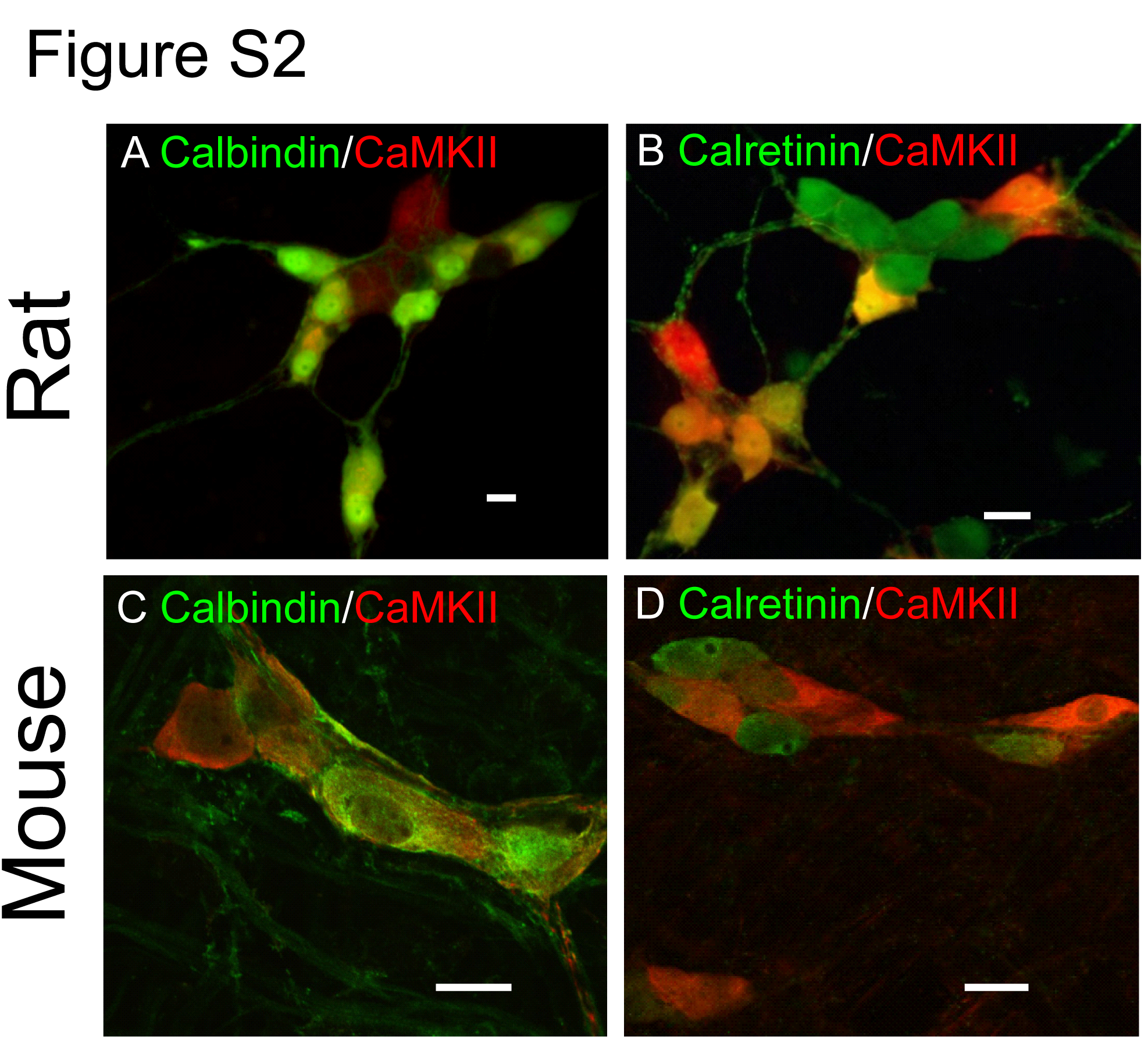

Supplement: Figure S2 — CaMKII-IR is co-locolized with both calbindin- (A, C) and calretinin-IR (B, D) in rat and mouse submucosal plexuses. (TIF) [file pone.0044426.s002.tif]

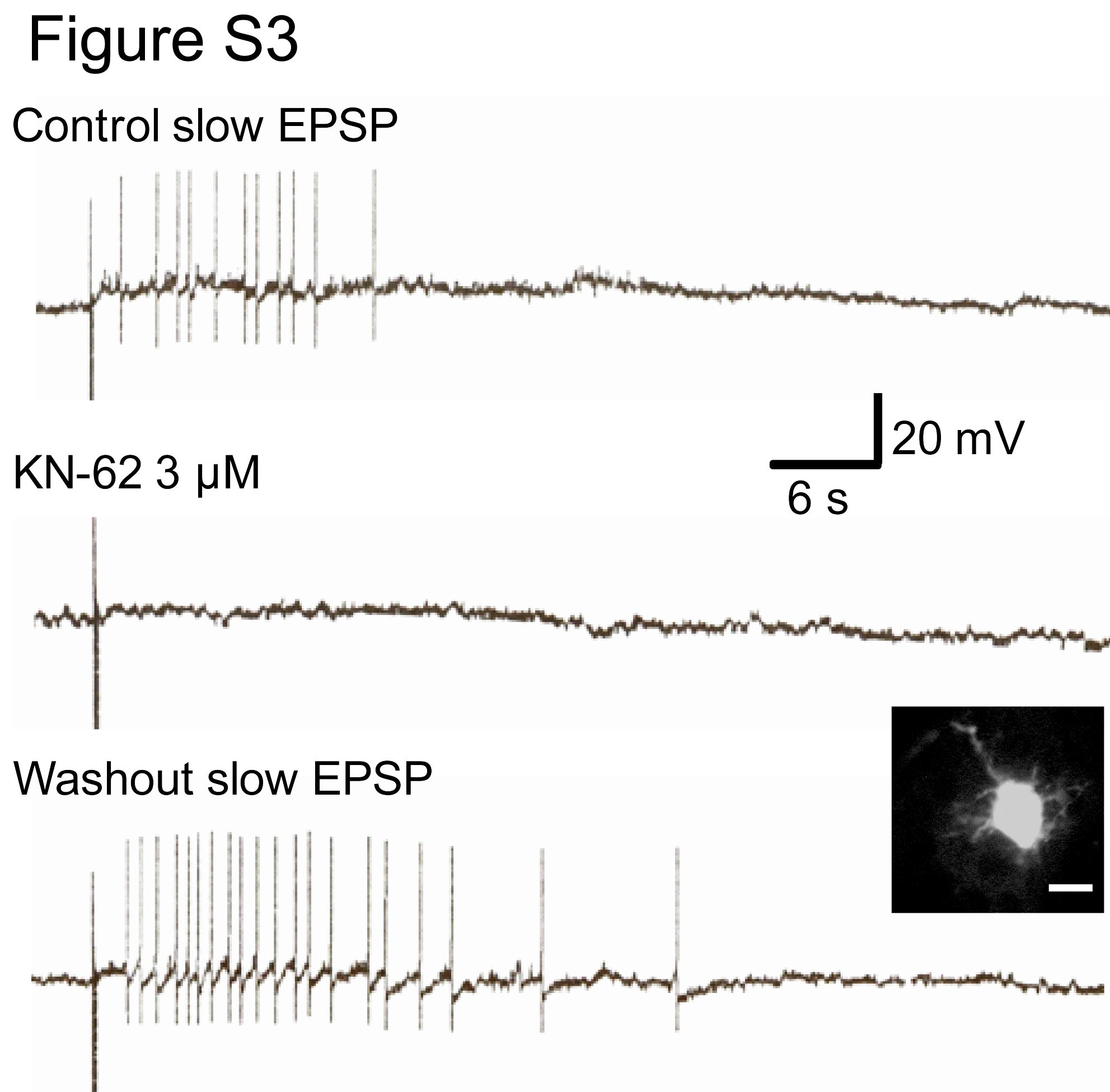

Supplement: Figure S3 — KN-62 suppresses slow EPSPs evoked by train stimulations (4 pulses, 150–200 Hz). KN-62 (3 µM) was applied through the bath solution. 30 min incubation of KN-62 suppressed the slow EPSP. The effect of KN-62 was reversible manner after a 40 min washout. The recorded neuron shows uniaxonal morphology as revealed by staining of biocytin injected via the recording microelectrode. Bar = 20 µm. (TIF) [file pone.0044426.s003.tif]

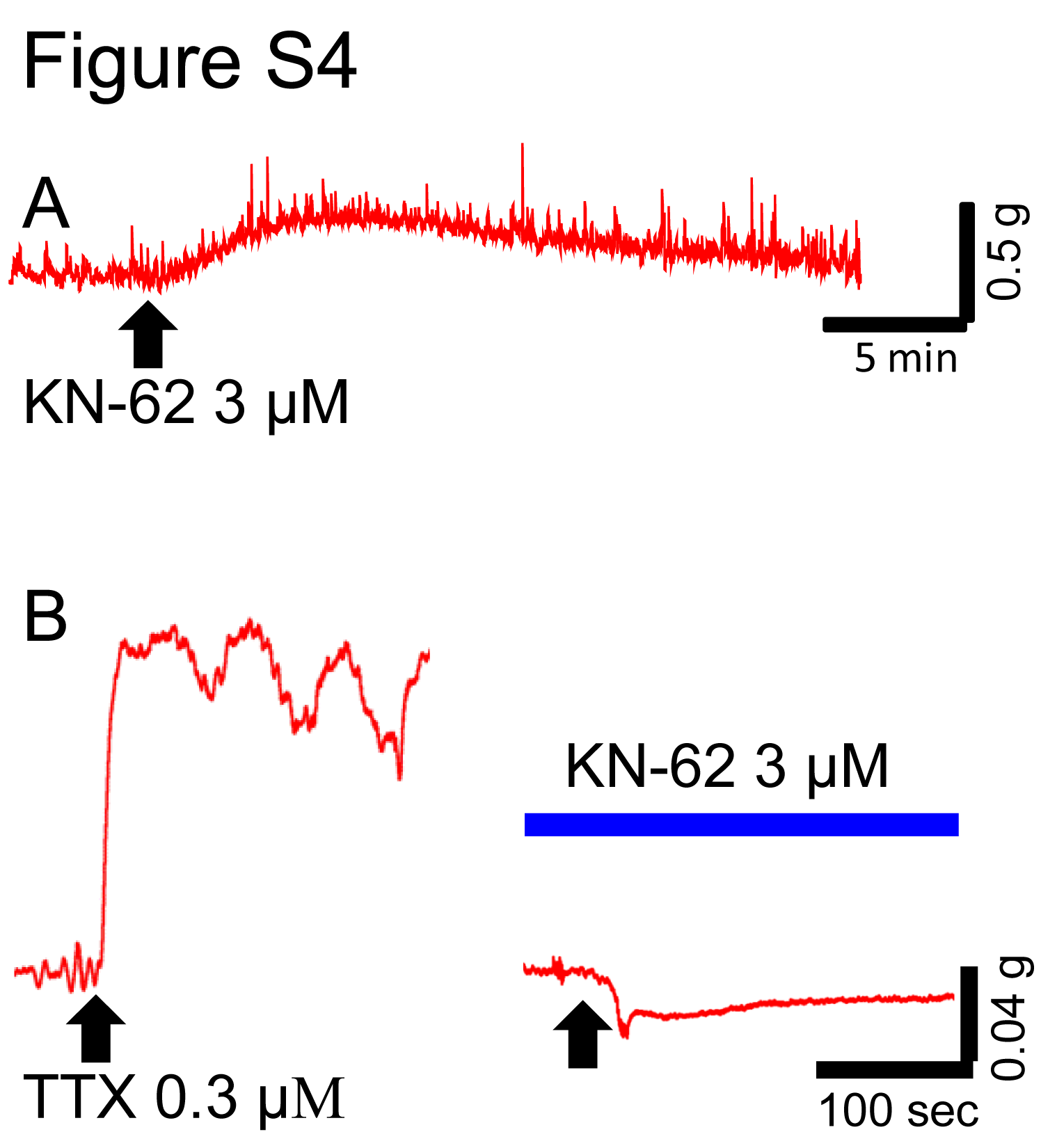

Supplement: Figure S4 — Application of KN-62 produces contraction or reverses TTX-induced contractile response in mouse colon strips. (A) Representative trace illustrates that KN-62 causes an increased contractile response in a mouse colon strip. (B) Application of KN-62 reversed TTX-induced contractile response in the same mouse colon strip. (TIF) [file pone.0044426.s004.tif]

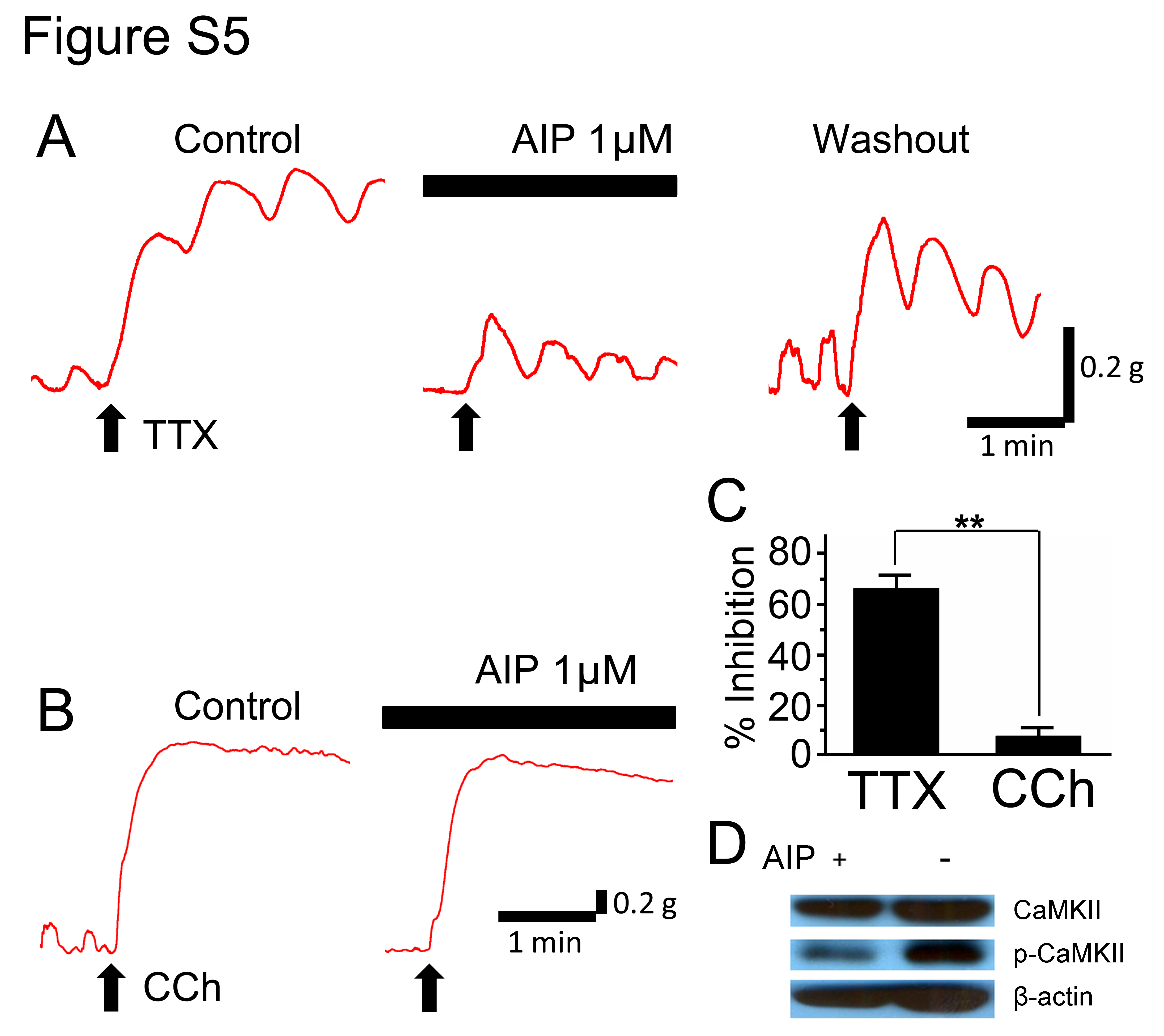

Supplement: Figure S5 — Suppression of TTX-induced contractile response by the selective CaMKII inhibitor, Autocamtide-2-related inhibitory peptide (AIP) in mouse colon. (A) AIP (1 µM) suppressed TTX-induced contractile response. The effect of AIP was partially reversible. (B) Effect of AIP on CCh-induced contraction in a colon strip. (C) The inhibitory effect of AIP on TTX-induced contractile response was significantly stronger than on CCh-induced contraction. ** p<0.01. (D) Representative Western Blot analysis shows that pretreatment with 1 µM AIP for 15 min substantially reduced the p-CaMKII-IR in the myenteric plexus preparations of mouse colon. The total CaMKII-IR was comparable between tissue preparations with and without treatment with KN-62. β-actin was used as a loading control. (TIF) [file pone.0044426.s005.tif]

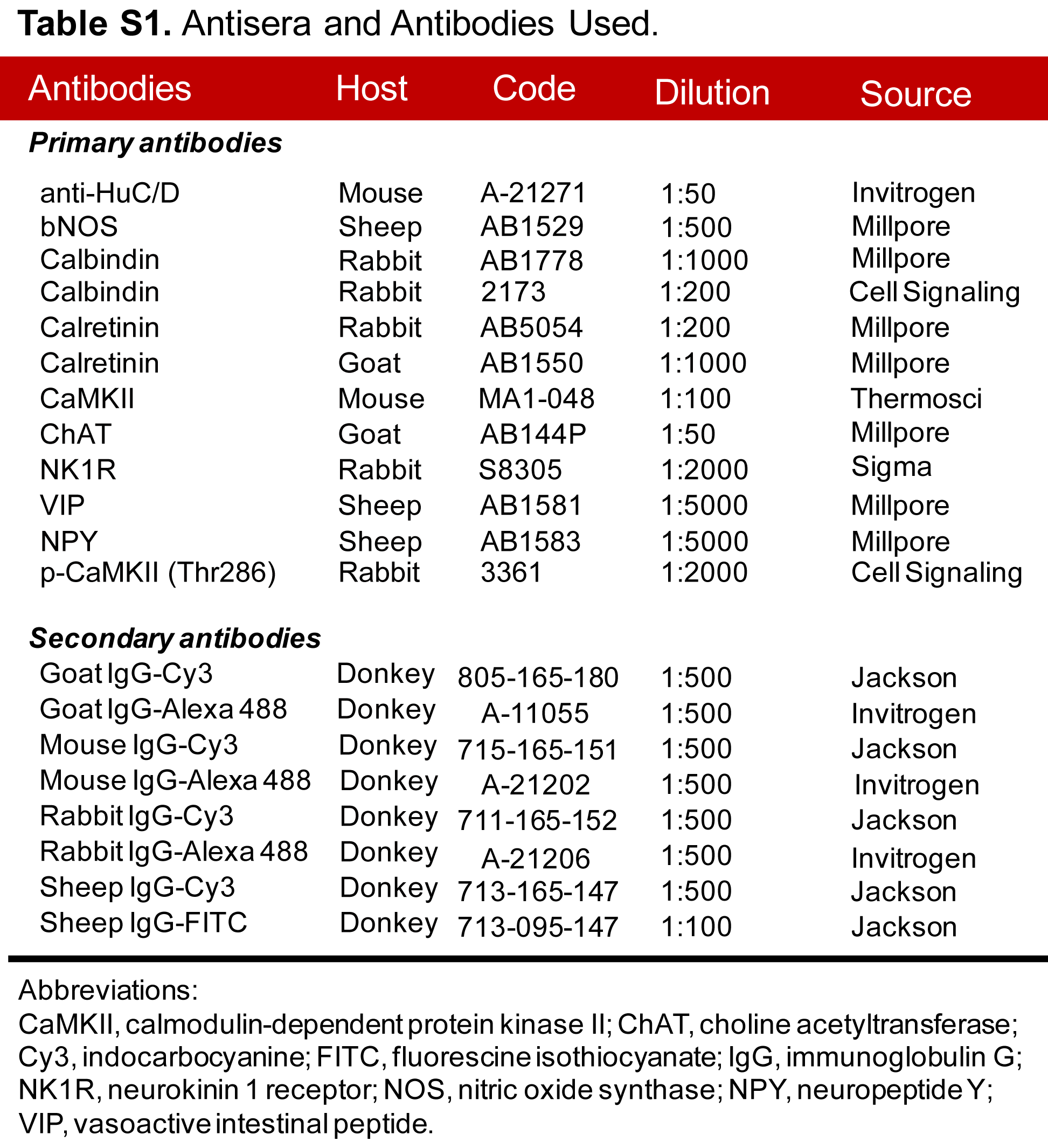

Supplement: Table S1 — Antisera and antibodies used. (TIF) [file pone.0044426.s006.tif]
