# Supplementary material for: CaMKII Is Essential for the Function of the Enteric Nervous System
Source: PLoS One. 2012 Aug 31;7(8):e44426. doi: 10.1371/journal.pone.0044426 (PMC3432132; doi:10.1371/journal.pone.0044426)
Supplement: Methods S1 — (DOCX) [file pone.0044426.s007.docx]

**Supplemental methods S1**

# Tissue Preparations

# Tissues were obtained from Hartley-Dawley guinea pigs, C57BL/6 mice and Sprague Dawley rats which were killed by decapitation under isofluorane inhalation. The procedures were approved by the Ohio State University Animal Experimentation Ethics Committee or The Center for Laboratory Animal Medicine and Care (CLAMC) at The University of Texas Health Science Center at Houston. Segments of gut were removed and placed in ice-cold Krebs’ solution that contained (in mM) NaCl, 120.9; KCl, 5.9; MgCl_2_, 1.2; NaH_2_PO_4_, 1.2; NaHCO_3_, 14.4; CaCl_2_, 2.5; and glucose, 11.5. The segments were cut along the mesenteric border and opened out. The tissue was stretched tautly and pinned, mucosa side up, in a Sylgard-covered petri dish. Tissue was immersed in Zamboni’s fixative (2% formaldehyde plus 0.2% picric acid in 0.1M sodium phosphate buffer, pH 7.0) for 3hr at room temperature. Following fixation, the tissue was washed (3×10min) in Dimethylsulphoxide (DMSO), followed by 3×10min washes in PBS. Whole mounts of the myenteric and submucous plexuses were prepared from these segments as described previously[^16^](#_ENREF_16).

# Fresh segments of human jejunum discarded during Roux-En-Y gastric bypass surgeries were used. Procedures for human studies were approved by the Institutional Biosafety Committee and The Office of Responsible Research Practices of The Ohio State University. Tissues of about 3 cm in length and 2 cm in width were stretched and pinned and fixed in Zamboni’s fixative overnight. The fixed tissues were either dissected for whole mount preparation or immersed in 30% sucrose overnight before preparations of 8 µm frozen sections.

**Western Blot**

Membrane proteins were extracted from guinea pig myenteric and submucosal plexuses of ileum and colon and mouse colonic myenteric plexus. Frozen specimens were crushed to powder by using a liquid nitrogen-cooled biopulverizer unit (Research Products International, Philadelphia, PA) and were homogenized in 350 μl lysing buffer (20 mM Tris-HCl, pH 7.5, 150 mM NaCl, 2 mM EDTA, 0.1% sodium dodecyl sulfate [SDS], 1% NP-40, 0.25% deoxycholate, 1 mM sodium orthovanadate, 1 mM PMSF, 1 mM NaF, with Complete Mini EDTA-free protease inhibitor cocktail tablet [Roche]). Homogenates were continually incubated in lysing buffer on ice for 1 hour and subsequently centrifuged at 10,000*g* for 20 minutes at 4°C to obtain the cell membrane fraction in the supernatant.

Protein levels were determined using Nanodrop (Bio-Rad), and 40 μg of protein per lane was resolved by gel electrophoresis followed by transfer to the nitrocellulose membrane (Amersham Pharmacia Biotech, Piscataway, NJ). Membranes were blocked with 5% nonfat milk in Tris-HCl-buffered saline with 0.1% Tween 20 (TBST) for 1 hour at room temperature. After being washed with TBS, the membranes were incubated overnight at 4°C with a primary antibody against CaMKII or p-CaMKII (Thr286) (Supplemental Table 1). The membranes were incubated for 1 hour at room temperature with horseradish peroxidase-conjugated goat anti-mouse IgG (1:20,000; Amersham) or goat anti-rabbit IgG (1:5,000; Amersham). The immunoblots were detected with enhanced chemiluminescence reagents (Amersham).

## Immunofluorescence

## Whole-mounts were incubated in 10% normal horse serum in PBS for 1hr at room temperature prior to exposure to primary antisera (see table 1), diluted in hypertonic PBS containing 10% normal horse serum, 0.3% Triton X-100 and 0.1% sodium azide. Tissue was placed in humidified chambers and processed for indirect single or double immunofluorescence staining by incubating the tissue for 18 hr at room temperature in a mouse anti-CaMKIIα antiserum (diluted 1:200) or mixture of primary antibodies (Supplemental Table 1) from different specifies for double labeling. After incubation with the primary antibodies, the tissue was washed (3×10min) in PBS, transferred to a humidified chamber and incubated at 37°C for 30min with a single or mixture of second antibodies conjugated with Alexa 488 or fluorescin isothiocyanate (FITC), and Cy3, diluted in hypertonic PBS containing 10% normal horse serum, 0.3% Triton X-100 and 0.1% sodium azide. Tissue was then rinsed in PBS as before, and coverslipped with Vectorshield (Vector, Burlingame, CA). For double-labeling of CaMKII and Anti-HuC/D (both of which were revealed by immunofluorescence using monoclonal antibodies), we proceeded immunofluorescent staining of CaMKII first with Cy3 labeling followed by anti-HuC/D labeling with Alexa 488. This is based on the fact that CaMKII is expressed by a subset of enteric neurons while anti-HuC/D is expressed by all enteric neurons.

## All preparations were examined with an epifluorescence microscope (Nikon Eclipse-1000) or Nikon A1 Confocal Laser Microscope System and analyzed using filter combination that enabled completely separate visualization of the different fluorophore with no interference from the others. Pictures were taken with a CCD digital camera and analyzed in a SPOT III program or NIS-Elements. The biocytin-injected neurons were revealed by fluorescin streptavidin. The preparations were cleared in three changes of DMSO and three 10min washes with phosphate-buffered saline. The preparations were reacted with fluorescin-labeled streptavidin (1:200) for 30min at 37°C and observed under an epifluorescence microscope (Nikon Eclipse-1000).

**Intracellular Recording**

Guinea pig submucosal preparations were prepared as described above and transferred to a Sylgard-covered petri dish superfused with warmed Krebs’ solution at 37°C and gassed with 95% O_2_ and 5% CO_2_, at a rate of 10-15 ml min^-1^. Transmembrane electric potentials were recorded with conventional intracellular microelectrodes filled with 4 % biocytin in 2 M KCl containing 0.05 M tris-(hydroxy-methyl)-aminomethane buffer (pH 7.4) and having resistances of 80-200 MΩ. The preamplifier (M767, World Precision Instruments, Sarasota, FL) had bridge circuitry for intraneuronal injection of electrical current. Constant current, rectangular pulses were driven by a Grass SD9 stimulator (Grass Instruments, Quincy, MA, USA). Electrometer output was amplified and observed on an oscilloscope (Tektronics 5113; Tektronics, Beaverton, OR, USA). All data were recorded on magnetic tape for later analysis. Synaptic potentials were evoked by focal electrical stimulation of interganglionic fiber tracts with electrodes made of 20 μm diameter Teflon-coated platinum wire and connected through a stimulus-isolation unit (Grass SIN 5) to a Grass S 48 stimulator. Chart records were monitored by an external force displacement transducer (Quantametrics, Newtown, PA) connected to a PowerLab data acquisition system (AD Instruments, Colorado Springs, CO). At the end of the experiments, neuronal tracer biocytin was injected into the impaled neurons from the recording electrodes by the passage of hyperpolarizing current (0.5 nA for 3-10 min). Then, the anal end of the preparation was marked and the tissue was transferred into a disposable chamber filled with fixative (2 % formaldehyde plus 15 % of a saturated solution of picric acid) and kept at 4°C overnight. All chemicals were purchased from Tocris Bioscience (Ellisville, MO, USA). Chemicals were applied by addition to the superfusion solution.

**Intestinal Motility Assay**

Intestinal contractile activity was determined as previously described.[^15^](#_ENREF_15) Briefly, 1-cm whole thickness strips from the colon of each mouse were prepared in the longitudinal direction and mounted in duplicate in 25-ml organ baths filled with Krebs-Ringer solution gassed with 5% CO_2_–95% O_2_. After a 30-minutes equilibration period, the isometric force was monitored by an external force displacement transducer (Quantametrics) connected to a PowerLab data acquisition system (AD Instruments). Mean contraction amplitude was used for quantification.

**Statistical Methods**

Statistically significant differences between means will be determined by paired *t*-tests or 2-tailed Student’s *t-*tests. P<0.05 is considered significantly different.
